# Association between metabolic syndrome and sexual dysfunction among men with clinically diagnosed diabetes

**DOI:** 10.1186/1758-5996-5-42

**Published:** 2013-07-29

**Authors:** Nafiu Amidu, William KBA Owiredu, Huseini Alidu, Charity Sarpong, Christian K Gyasi-Sarpong, Lawrence Quaye

**Affiliations:** 1 Department of Biomedical Laboratory Science, School of Medicine and Health Sciences, University for Development Studies, Tamale, Ghana; 2 Department of Molecular Medicine, School of Medical Sciences, College of Health Sciences, Kwame Nkrumah University of Science and Technology, Kumasi, Ghana; 3 Tema General Hospital, Tema, Greater Accra Region, Ghana; 4 Department of Surgery, (Urology Unit) Komfo Anokye Teaching Hospital/College of Health Sciences, Kwame Nkrumah University of Science and Technology, Kumasi, Ghana

## Abstract

**Background:**

The worldwide epidemic of diabetes and obesity has resulted in a rapid upsurge in the prevalence of metabolic syndrome (MetS). MetS makes the individual liable to endothelial dysfunction which can initiate sexual dysfunction (SD). This study assessed the association between MetS and SD among clinically diagnosed diabetic subjects in Tema, Greater Accra Region of Ghana.

**Method:**

Sexual functioning was assessed using Golombok Rust Inventory of Sexual Satisfaction in 300 consecutive diabetic men visiting the diabetic clinic of Tema General Hospital between November, 2010 and March, 2011. Anthropometric data including waist and hip circumference as well as blood pressure were measured. The levels of fasting blood glucose and serum lipid profile were assessed. All the men had a steady heterosexual relationship for at least 2 years before enrolment in the study.

**Results:**

The response rate was 91.3% out of the 300 subjects recruited for the study. Those with SD were significantly older and had diabetes for a longer period as compared to those without SD. The prevalence of MetS as defined by the various criteria was 78.8%, 43.4% and 51.8% for WHO, NCEP ATP III and IDF respectively. Central obesity (p = 0.0482) and raised blood pressure (p = 0.0309) are the significant MetS components when the studied population was stratified according to sexual functioning. Generally, SD as well as its sub-scales correlate positively with age, blood pressure, duration of diabetes and MetS score. Whereas TC and LDL-c correlated positively with non-communication, TG correlates positively with avoidance and infrequency.

**Conclusion:**

SD and its sub-scales have a direct relationship with duration of diabetes, blood pressure and MetS score from this study. Central obesity and raised blood pressure seem to be the link between MetS and SD among this clinically diagnosed diabetic subjects.

## Background

Reduced sexual function, defined as the inability to participate in a sexual relationship as one would wish has multifactorial causes with medical and psychological complications of diabetes being implicated [1-3]. By 2025, approximately 380 and 322 million people have been projected to develop diabetes and sexual dysfunction (SD) respectively [4]. Metabolic syndrome (MetS), diabetes, cardiovascular disease (CVD), obesity and SD were previously perceived as independent factors for aging men. However, emerging data gives an indication of their interrelatedness via multiple mechanisms [5]. MetS and SD are conditions associated with aging and account for reduced quality of life. In the face of a growing worldwide epidemic of obesity, diabetes is expected to facilitate the progression of MetS and SD and thus lead to a reduction in quality of life.

MetS is defined as a constellation of conditions involving at least three out of five risk factors including abdominal obesity, elevated blood pressure, elevated fasting blood glucose, high triglyceride as well as low HDL-cholesterol. The prevalence of MetS and SD would vary depending on the defining criteria, age, genetic make-up, diet, occupation, cultural background, socioeconomic status, medical condition as well as the population involved. Depending on the criteria used among Ghanaians, the prevalence of MetS varies between 11.5 - 15.5% among psychiatric patients [6], 1.6 – 14.4% among active sportsmen and women [7], 13 – 18% among garage workers in the automobile industry [8] and 30.1% among chronic kidney disease patients [9]. MetS affected individuals are subjected to endothelial dysfunction and autonomic hyperactivity which sets the stage for metabolic risk factors inducing SD [10].

The prevalence of SD among the Ghanaian community has been reported to vary between 59.8 to 70% depending on the population [11,12], medical conditions [13] and marital status [14]. Previous reports among this same population of clinically diagnosed diabetic subjects indicated an SD rate of 69.3% [15]. This rate appears to be related to infrequency of the sexual act (79.2%), non-sensuality (74.5%), dissatisfaction (71.9%), lack of communication (70.8%) and impotence (67.9%). Other areas of sexual function, including premature ejaculation (56.6%) and avoidance (42.7%) were also substantially affected [15].

Even though some studies support the existence of an association between CVD and MetS which is a risk factor for endothelial dysfunction as well as vascular damage, a link between SD and MetS also appear to exist. However, the nature of the association using Golombok Rust Inventory of Sexual Satisfactory as an assessment of sexual function with MetS is limited among diabetic subjects. These are interconnected conditions in terms of their cause and require an integrated approach. This study attempts to define the association between SD and MetS among clinically diagnosed diabetic subjects using Golombok Rust Inventory of Sexual Satisfactory. To our knowledge, this is the first study seeking to determine the association between MetS and SD among this population in Ghana.

## Methods

### Participants

This cross-sectional study was conducted among 300 clinically diagnosed diabetic patients visiting Tema General Hospital in the Greater Accra region of Ghana. Sexually active individuals who are at least 18 years and engaged in or maintained heterosexual relationship for at least 2 years regardless of their marital status were recruited in a consecutive procedure from November 2010 to March 2011. Participation of the respondents was voluntary and informed consent was obtained from each participant. The study was approved by the Committee on Human Research, Publication and Ethics of the School of Medical Science and the Komfo Anokye Teaching Hospital, Kumasi.

### Anthropometric data

Information on age and duration of diabetes were collected using a semi-structured questionnaire. Waist circumference (to the nearest centimetre) was measured with a Gulick II spring-loaded measuring tape (Gay Mill, WI) midway between the inferior angle of the ribs and the suprailiac crest. Hip circumference was measured as the maximal circumference over the buttocks in centimetres and the waist-to-hip ratio (WHR) calculated by dividing the waist circumference (cm) by the hip circumference (cm). Blood pressure was measured by qualified nurses using a mercury sphygmomanometer and stethoscope. Measurements were taken from the upper left arm after subjects had been sitting for >5 minutes in accordance with the recommendation of the American Heart Association [16]. Duplicate measurements were taken with a 5 minutes rest interval between measurements and the mean value was recorded to the nearest 2.0 mmHg.

### Golombok rust inventory of sexual satisfaction (GRISS)

Sexual function was assessed using GRISS questionnaire. The GRISS has 28 items on a single sheet and is used for assessing the existence and severity of sexual problems in heterosexual couples or individuals who have a current heterosexual relationship. All the 28 questions are answered on a five-point (Likert type) scale from “*always*”, through “*usually*”, “*sometimes*”, and “*hardly ever*”, to “*never*”. It provides overall scores of the quality of sexual functioning within a relationship. In addition, subscale scores of impotence, premature ejaculation, infrequency, non-communication, dissatisfaction, non-sensuality and avoidance can be obtained and represented as a profile. Responses are summed up to give a total raw score (range 28–140). The total score and subscale scores are transformed using a standard nine point scale, with high scores indicating greater problems. Scores of five or more are considered to indicate SD. The GRISS was chosen because it is standardized, easy to administer and score, relatively unobtrusive and substantially inexpensive. The reliability of the overall scales has been found to be 0.94 for men, and that of the subscales on average 0.74 (ranging between 0.61 and 0.83). Validity has been demonstrated under a variety of circumstances [17-19].

### Sample collection, preparation and analysis

After observing an overnight fast, 6 ml of venous blood sample was collected from each participant in the morning between 07:00 to 09:00 GMT into fluoride oxalate and evacuated gel tubes (Becton Dickinson, Rutherford, NJ). Blood samples in the fluoride oxalate and evacuated gel tubes were centrifuged at 500 *g* for 15 minutes within 30 minutes of sample collection and separated into plasma and serum respectively. The plasma sample was used for fasting blood glucose measurement using BT 3000® Random Access Chemistry Analyzer (Biotecnica, Italy). Serum derived from the evacuated gel tubes were stored in cryovials at −80°C until time for lipid profile measurement using BT 3000® Random Access Chemistry Analyzer (Biotecnica, Italy). The methods adopted by the automated instruments for the determination of biochemical parameters were according to the reagent manufacturers’ instructions (JAS Diagnostics, Inc. Miami Florida, USA and Abbott Diagnostics, USA).

### Metabolic syndrome definitions

#### National cholesterol education program, adult treatment panel III (NCEP ATP III)

MetS was defined according to the criteria of the National Cholesterol Education Program, Adult Treatment Panel III (NCEP ATP III) to include individuals with three or more of the following five components: (1) abdominal obesity (waist circumference > 102 cm); (2) high TG ≥ 1.7 mmol/L (150 mg/dl); (3) low HDL-C : < 0.9 mmol/L (< 40 mg/dl); and (4) High BP (systolic BP ≥ 130 mm Hg or diastolic BP ≥ 85 mm Hg or treatment of hypertension) and (5) high fasting glucose ≥ 6.1 mmol/l [20].

#### International Diabetes Federation (IDF)

According to the new definition by the International Diabetes Federation (IDF) [21], MetS can be diagnosed if central obesity (waist measurement >90 cm) is accompanied by any 2 of the following 4 factors: (1) TG levels of 1.7 mmol/L or greater, (2) an HDL cholesterol lower than 1.03 mmol/L, (3) a blood pressure (BP) of 130/85 mm Hg or greater or treatment of previously diagnosed hypertension, and (4) a fasting blood glucose (FBG) of 5.6 mmol/L or greater or previously diagnosed type 2 diabetes.

#### World Health Organization (WHO)

WHO criteria [21] required presence of diabetes mellitus, impaired glucose tolerance or insulin resistance and any two of the following: (1) waist-to-hip ratio >0.90, (2) blood pressure ≥140/90 mmHg or on medication, (3) diabetes ≥6.1 mmol/L or on medication for diabetes, impaired glucose tolerance or insulin resistance, (4) triglyceride ≥1.7 mmol/L and/or HDL-C <0.91 mmol/L.

### Statistical analysis

The data were presented as mean ± standard deviation or percentages. Continuous data were analyzed using unpaired *t*-tests whilst categorical variables were analyzed using Fischer’s exact test. In all statistical tests, a value of *p* < 0.05 was considered significant. All analyses were performed using SigmaPlot for Windows, Version 11.0, (Systat Software, Inc. Erkrath, Germany) www.systat.com.

## Results

This study indicated 91.3% response rate out of the 300 subjects that were recruited for the study. Twenty subjects found the subject matter too sensitive and hence refused to partake in the study and 6 of the respondents returned incomplete data leaving 274 evaluable data. As shown in Table 1, respondents with SD were significantly older and had been diagnosed with diabetes for a longer period compared to the respondents without SD. The mean ages as well as the mean duration of diabetes from this study were 59.9 ± 11.3 years and 6.8 ± 5.9 years respectively. Those with SD also had significantly higher level of mean SBP as compared to their counterparts without SD. The mean ± standard deviation of the biochemical parameters was not significantly different between those with and without SD, even though those with SD had generally higher values (Table 1). The mean raw score for SD and its sub-scales were significantly higher among those with SD except for avoidance and infrequency (Table 1).

**Table 1:** General characteristic of the study population stratified by sexual dysfunction

**Variables**	**Total (n = 274)**	**NSD (n = 84)**	**SD (n = 190)**	**P value**
***Socio*-*demographic data***				
Age (years)	59.9 ± 11.3	56.2 ± 11.6	65.1 ± 7.5	<0.0001
Duration of diabetes (years)	6.8 ± 5.9	6.0 ± 5.6	8.5 ± 6.2	0.0012
***Anthropometric data***				
SBP (mmHg)	151.5 ± 24.7	148.8 ± 25.7	157.6 ± 21.1	0.0063
DBP (mmHg)	96.2 ± 17.7	94.8 ± 16.9	99.4 ± 19.1	0.0504
Hip circumference (cm)	100.7 ± 9.5	100.0 ± 7.6	101.0 ± 10.3	0.4559
Waist circumference (cm)	94.5 ± 16.0	94.6 ± 16.3	94.4 ± 15.9	0.9025
WHR	0.9 ± 0.2	0.9 ± 0.2	0.9 ± 0.1	0.5362
***Biochemical parameters***				
FBG (mmol L^-1^)	9.4 ± 4.0	9.3 ± 3.4	9.4 ± 4.2	0.8254
Total cholesterol (mmolL^-1^)	5.2 ± 1.2	5.1 ± 1.1	5.3 ± 1.3	0.2021
Triglyceride (mmolL^-1^)	1.4 ± 0.7	1.4 ± 0.8	1.5 ± 0.7	0.3137
HDL-cholesterol (mmolL^-1^)	1.2 ± 0.3	1.2 ± 0.3	1.2 ± 0.3	0.5161
LDL-cholesterol (mmolL^-1^)	3.3 ± 1.1	3.3 ± 1.0	3.5 ± 1.2	0.1279
Testosterone (ng mL^-1^)	6.3 ± 2.5	6.7 ± 2.8	6.0 ± 2.1	0.0250
***Raw score for sexual dysfunction and its sub*-*scales***				
Sexual dysfunction	72.2 ± 10.6	58.8 ± 8.0	78.1 ± 4.4	<0.0001
Impotence	11.3 ± 2.1	9.2 ± 1.6	12.3 ± 1.6	<0.0001
Premature ejaculation	7.4 ± 2.9	4.8 ± 1.5	8.5 ± 2.6	<0.0001
Non-sensuality	11.6 ± 2.5	9.2 ± 2.2	12.7 ± 1.7	<0.0001
Avoidance	8.0 ± 3.2	7.8 ± 4.5	8.1 ± 2.5	0.5710
Dissatisfaction	11.3 ± 1.6	10.0 ± 1.7	11.8 ± 1.2	<0.0001
Non-communication	4.2 ± 1.5	3.0 ± 1.1	4.8 ± 1.3	<0.0001
Infrequency	5.3 ± 1.4	5.1 ± 1.4	5.4 ± 1.4	0.0796

Data are presented as mean ± s.d; P-value defines the level of significance when subjects with no sexual dysfunction (NSD) were compared to those with sexual dysfunction (SD)(Unpaired *t*-test); *SBP*-systolic blood pressure; *DBP*-diastolic blood pressure; *WHR*-waist to hip ratio; *FBG*-fasting blood glucose; *HDL*-high density lipoprotein; *LDL*-low density lipoprotein.

The prevalence of MetS as defined by the various criteria was 78.8%, 43.4% and 51.8% for WHO, NCEP ATP III and IDF respectively. Even though the prevalence of MetS was found to be higher among those with SD irrespective of the criteria used, the differences were not significant (Table 2). About 80%, 40% and 60% of the studied population had MetS score of 3 or more using WHO, NCEP ATP III and IDF criteria respectively. Classification according to sexual function indicated that those with SD generally have lower rates of MetS score of 1 and 2 but higher rates of MetS score of 3 or more notwithstanding the fact that the differences were not significant (Table 2).

**Table 2:** Prevalence of metabolic syndrome and metabolic score among the studied population stratified by sexual function

**Variable**	**Total (n = 274)**	**NSD (n = 84)**	**SD (n = 190)**	**P value**
***Prevalence of MetS***				
WHO	216(78.8%)	64(76.2%)	152(80.0%)	0.4766
NCEP ATP III	119(43.4%)	35(41.7%)	84(44.2%)	0.6953
IDF	142(51.8%)	43(51.2%)	99(52.1%)	0.8889
***Prevalence of clustering of components of MetS***				
**WHO**				
0	0(0.0%)	0(0.0%)	0(0.0%)	
1	11(4.0%)	4(4.8%)	7(3.7%)	0.6752
2	46(16.8%)	16(19.0%)	30(15.8%)	0.5059
≥3	217(79.2%)	64(76.2%)	153(80.5%)	0.4149
**NCEP ATP III**				
0	3(1.1%)	0(0.0%)	3(1.6%)	0.2469
1	46(16.8%)	13(15.5%)	33(17.4%)	0.6992
2	107(39.1%)	34(40.5%)	73(38.4%)	0.7478
≥3	118(43.1%)	32(38.1%)	86(45.3%)	0.2692
**IDF**				
0	1(0.4%)	0(0.0%)	1(0.5%)	0.5053
1	20(7.2%)	8(9.5%)	12(6.3%)	0.3466
2	87(31.8%)	31(36.9%)	56(29.5%)	0.2231
≥3	166(60.6%)	49(58.3%)	117(61.6%)	0.6122

Data are presented as proportions; P value defines the level of significance when subjects with no sexual dysfunction (NSD) were compared to those with sexual dysfunction (SD) (Fischer’s exact test).

Using the WHO criteria, the highest prevalence of components of MetS was central obesity as measured by WHR (76.6%), followed by hypertension (59.5%) and hyperlipidemia (47.8%). From the NCEP ATP III criteria, the highest prevalence of components of MetS was raised BP (73.0%) followed by raised TG (32.1%), reduced HDL-C(i.e. 28.5%) and abdominal obesity as measured by WC (19.3%). Raised BP had the highest prevalence rate (73.0%), followed by abdominal obesity (65.0%), raised TG (32.1%) and reduced HDL-C (28.5%) using the IDF criteria (Table 3). When the studied population was classified according to sexual functioning, those with SD significantly (p = 0.0482) had higher rates of central obesity (80.0%) as compared to those without SD (69.9%) using WHO criteria. However, when the NCEP ATP III and IDF criteria were applied, the prevalence rate of MetS among individuals with raised BP was significantly (p = 0.0309) higher among those with SD (76.8%) as compared to those without SD (64.3%) as shown in Table 3.

**Table 3:** Prevalence of the various metabolic syndrome risk factors among the study population classified by sexual function

**Variable**	**Total (n = 274)**	**NSD (n = 84)**	**SD (n = 190)**	**P value**
**WHO**				
Central Obesity – WHR	210(76.6%)	58(69.0%)	152(80.0%)	0.0482
Raised fasting glucose	274(100.0%)	84(100.0%)	190(100.0%)	
Hyperlipidaemia	131(47.8%)	36(42.9%)	95(50.0%)	0.2751
Raised blood pressure	163(59.5%)	47(56.0%)	116(61.1%)	0.4278
**NCEP ATP III**				
Abdominal Obesity – WC	53(19.3%)	13(15.5%)	40(21.1%)	0.2813
Raised fasting glucose	274(100.0%)	84(100.0%)	190(100.0%)	
Raised Triglyceride	88(32.1%)	25(29.8%)	63(33.2%)	0.5788
Raised blood pressure	200(73.0%)	54(64.3%)	146(76.8%)	0.0309
Reduced HDL-C	78(28.5%)	21(25.0%)	57(30.0%)	0.3978
**IDF**				
Abdominal Obesity – WC	178(65.0%)	52(61.9%)	126(66.3%)	0.4804
Raised fasting glucose	274(100.0%)	84(100.0%)	190(100.0%)	
Raised Triglyceride	88(32.1%)	25(29.8%)	63(33.2%)	0.5788
Raised blood pressure	200(73.0%)	54(64.3%)	146(76.8%)	0.0309
Reduced HDL-C	78(28.5%)	21(25.0%)	57(30.0%)	0.3978

Data are presented as proportion with corresponding percentages in parenthesis. The proportions were compared using Fischer’s exact test.

Generally, SD as well as its sub-scales correlated positively with age, blood pressure and duration of diabetes. Whereas TC and LDL-c correlated positively with non-communication, TG correlated positively with avoidance and infrequency (Table 4). Sexual dysfunction, PE, NS, AV, DIS and INF generally correlated positively with MetS scores with small size effect irrespective of the criteria used (Table 4).

**Table 4:** Partial correlations between sexual dysfunction parameters and determinants of metabolic syndrome

**Variable**	**SD**	**IMP**	**PE**	**NS**	**AV**	**DIS**	**NC**	**INF**
Age	0.39***	0.31***	0.33***	0.39***	0.15*	0.24***	0.21***	−0.06
SBP	0.23***	0.15*	0.27***	0.23***	0.02	0.22***	0.08	0.05
DBP	0.19**	0.15*	0.21***	0.17**	−0.01	0.24***	0.06	0.09
DOD	0.26***	0.11	0.27***	0.20***	0.00	0.27***	0.09	0.05
WC	0.04	0.11	−0.04	−0.10	−0.03	−0.09	−0.08	−0.05
WHR	0.01	0.08	−0.05	0.03	0.10	0.03	0.13*	0.02
FBG	0.06	0.05	0.04	0.04	0.16**	−0.07	0.00	0.09
TC	0.05	0.05	−0.02	−0.04	0.01	−0.03	0.18**	−0.04
TG	0.14	0.04	−0.09	−0.02	0.23***	−0.03	0.03	0.29***
HDL-c	0.05	0.11	0.03	0.06	0.00	0.03	0.03	0.02
LDL-c	0.06	0.00	−0.02	−0.09	0.10	−0.04	0.18**	0.01
WHO	0.16**	0.08	0.17**	0.16**	0.10	0.15*	0.00	0.15*
ATP	0.16**	0.01	0.13*	0.12*	0.14*	0.11	0.08	0.19**
IDF	0.21***	0.08	0.17**	0.22***	0.14*	0.16**	0.06	0.16**

*Correlation is significant at the 0.05 level (2-tailed), **Correlation is significant at the 0.01 level (2-tailed), ***Correlation is significant at the 0.001 level (2-tailed). Boldface r = Pearson product moment correlation coefficient with a medium size (0.30 ≤ r ≥ 0.50) effect. *SBP*-systolic blood pressure, *DBP*-diastolic blood pressure, *DOD*-duration of diabetes, *WC*-waist circumference, *WHR*-waist to hip ratio, *TC*-total cholesterol, *TG*-triglycerides, *HDL*-c-high density lipoprotein-cholesterol, *LDL*-c-low density lipoprotein-cholesterol, *SD*-sexual dysfunction, *IMP*-impotence, *PE*-premature ejaculation, *NS*-non-sensuality, *AV*-avoidance, *DIS*-dissatisfaction, *NC*-non-communication, *INF*-infrequency.

## Discussion

Even though the causal relationship is still unclear, available data supports an association between SD and MetS. Thus, this study was conducted to define the association between SD and MetS among men who have been clinically diagnosed with diabetes mellitus. The prevalence of SD has been reported among these same clinically diagnosed diabetic men to be 69.3% [15]. This SD rate was found to be related to infrequency (79.2%), non-sensuality (74.5%), dissatisfaction with sexual acts (71.9%), non-communication (70.8%) and impotence (67.9%). Other areas of sexual function, including premature ejaculation (56.6%) and avoidance (42.7%) were also substantially affected [15]. SD was found to be associated with decreased testosterone levels and varied according to age with obesity being one of the determinants [15].

Irrespective of the criteria used in the definition, MetS does not represent a disease state in itself but a step towards global dysfunction. From this study, ≈ 40% to 80% of the diabetic men can be classified as having MetS depending on the definitive criteria employed. Among those with SD, the prevalence of MetS was 44.2%, 52.1% and 80.0% using the NCEP ATP III, IDF and WHO criteria respectively. The prevalence of MetS observed in this study is similar to the 90.1% observed among men with SD [22] and the 96.5% of MetS subjects exhibiting SD [23]. The lack of significant difference in the estimated prevalence of MetS between those with and without SD from this study could be due to the fact that all the subjects were diabetic and are thus susceptible to the development of endothelial dysfunction since both SD and MetS are thought to be mediated by endothelial dysfunction.

However, the positive correlation between SD and its sub-scales with MetS scores lends credence to the association between MetS and SD. MetS could lead to SD through various processes. MetS is associated with decreased testosterone level and can lead to SD through endothelial dysfunction which has been linked with vascular disorders or change in testosterone:estrogen levels [24] or through atherosclerosis which can cause structural damage within the penile tissues [25].

Among these diabetic subjects, a positive correlation existed between SD, premature ejaculation, non-sensuality and dissatisfaction with duration of diabetes. In diabetic subjects, there is on-going cellular activity leading to increased production of reactive oxygen species (e.g. superoxide anion) that deactivates nitric oxide [26,27]. Increased endothelial dysfunction among diabetic subjects also leads to reduction in vascular nitric oxide, impaired vasodilation, increased free radical production and atherosclerotic damage [28]. Long-standing diabetes can also lead to glycation of penile cavernosal tissue [29] and urogenital sensory neuropathy [30].

MetS, diabetes, CVD including hypertension, obesity and SD were previously thought to be independent factors for aging men. However, MetS and SD have received a great deal of attention over the past decades due to their association with CVD and diabetes in the face of a growing worldwide epidemic of obesity. The complex relationship between MetS and SD among these diabetic subjects could be explained by the prevailing central obesity and raised blood pressure. Obesity is not only a major component of MetS, but also a key factor for MetS progression. Increased central obesity normally leads to increased leptin that could be responsible for the decrease in testosterone level previously reported among this population [15] via functional leptin receptor isoform on Leydig cells [31]. Enhanced aromatase activity also irreversibly catalyses the conversion of testosterone to oestradiol due to the central obesity of MetS leading to further visceral adiposity, increased aromatase function and a positive feedback loop (i.e. hypogonadal-obesity cycle) [32,33].

The mediating effect of raised blood pressure between MetS and SD is in conformity with previous studies [34,35]. Increased sympathetic tone in raised blood pressure combined with endothelial dysfunction in MetS, or other organic status, are the most plausible pathway for inducing SD. The role of lipid profile including TC, LDL-c and TG cannot be ignored due to their positive correlation with non-communication, avoidance and infrequency which are key determinants of sexual functioning in an individual. In MetS subjects, increased production of cholesterol, LDL-c and TG can lead to destruction of endogenous nitric oxide synthetase [36,37], decreased bioavailability of NO, increase in vasoconstrictor factors and hence damaged endothelium-based vasodilatation at the early stages of atherosclerosis [38,39]. Further scientific enquiry is required for the establishment of a reciprocal cause between MetS and SD among diabetic male patients.

## Conclusion

The prevalence of MetS as defined by the various criteria was 78.8%, 43.4% and 51.8% for WHO, NCEP ATP III and IDF respectively. The prevalence of MetS amongst subjects with SD was 80.0%, 44.2% and 52.1% using WHO, NCEP ATP III and IDF criteria respectively. There was a strong positive correlation of SD and its sub-scales with MetS scores, blood pressure, and the duration of diabetes which implies the worsening of SD once a subject had MetS. Central obesity and raised blood pressure are the link between MetS and SD among this cohort of diabetic patients.

## Competing interests

The authors declare that they have no competing interests.

## Authors’ contributions

NA and WKBAO developed the concept and designed the study. NA, WKBAO, HA, CS, LQ and CKG-S administered the questionnaire, measured anthropometric parameters and assay for FBS, Lipid profile and testosterone, analysed and interpreted the data. NA, HA, CS, LQ and CKG-S drafted the manuscript. NA, WKBAO, HA, CS, LQ and CKG-S revised the manuscript for intellectual content. All authors read and approved the final manuscript.
